# Knowledge of obstetric danger signs among recently-delivered women in Chamwino district, Tanzania: a cross-sectional study

**DOI:** 10.1186/s12884-017-1469-3

**Published:** 2017-08-29

**Authors:** Deogratius Bintabara, Rose N. M. Mpembeni, Ahmed Abade Mohamed

**Affiliations:** 1 Department of Public Health, College of Health Sciences, The University of Dodoma, P.O Box 259, Dodoma, Tanzania; 2 Department of Global Health Entrepreneurship, Division of Public Health, Graduate School of Tokyo Medical and Dental University, 1-5-45 Yushima, Bunkyo-ku, Tokyo, 113-8519 Japan; 3 Department of Epidemiology and Biostatistics, Muhimbili University of Health and Allied Sciences, P.O. Box 65015, Dar Es Salaam, Tanzania; 4 Tanzania Field Epidemiology and Laboratory Training Programme (TFELTP), Dar Es Salaam, Tanzania

**Keywords:** Obstetric danger signs, Skilled birth attendants, Chamwino district, Tanzania

## Abstract

**Background:**

Low knowledge of danger signs has been shown to delay seeking obstetric care which leads to high maternal mortality and morbidity worldwide. In Tanzania about half of pregnant women are informed about obstetric danger signs during antenatal care, but the proportion of those who have full knowledge of these obstetric danger signs is not known. This study assessed the knowledge of obstetric danger signs and its associated factors among recently-delivered women in Chamwino District, Tanzania.

**Methods:**

A community-based cross-sectional study was conducted in January 2014 in Chamwino District, Tanzania. A woman was considered knowledgeable if she spontaneously mentioned at least five danger signs in any of the three phases of childbirth (pregnancy, childbirth and postpartum) with at least one in each phase. Multistage cluster sampling was used to recruit study participants. Descriptive and bivariate analyses were conducted. Multivariable logistic regression analyses were performed to control for confounding and other important covariates.

**Results:**

A total of 428 women were interviewed. The median age (IQR) was 26.5 (22–33) years. Only 25.2% of respondents were knowledgeable about obstetric danger signs during pregnancy, childbirth/labour and postpartum. Significant explanatory variables of being knowledgeable about obstetric danger signs were found to be maternal education (AOR = 1.96; 95% CI: 1.01, 3.82), maternal occupation (AOR = 2.23; 95% CI; 1.10, 4.52), spouse occupation (AOR = 2.10; 95% CI: 1.02, 4.32) and counseling on danger signs (AOR = 3.42; 95% CI: 1.36, 8.62) after controlling for the clustering effect, confounding and important covariates.

**Conclusion:**

A low proportion of women was found to be knowledgeable about obstetric danger signs in Chamwino district. Therefore, we recommend the Ministry of Health to design and distribute the maternal health booklets that highlight the obstetric danger signs, and encourage antenatal care providers and community health workers to provide frequent health education about these danger signs for every pregnant woman in order to increase their level of knowledge about obstetric danger signs.

## Background

High maternal mortality ratio (MMR) is a cause of concern for a number of countries worldwide [1]. In 2010, global MMR was 210 maternal deaths per 100,000 live births this hasdeclinedfrom400 maternal death per 100,000 live births reported in the 1990s [2]. Despite this global achievement, MMR continues to be a major public health challenge in developing countries where MMR can be up to 15 times higher than that in developed countries [2, 3]. Tanzania is among the developing countries with high MMR which is estimated to be 556 maternal deaths per 100,000 live births in 2015–16. This ratio is higher compared to 454 maternal deaths per 100,000 live births reported in 2010 [4, 5]. Most causes of maternal mortality are preventable and attributed to three delays: delay in the decision to seek care, delay in reaching the place of care, and delay in receiving appropriate care [6]. Poor knowledge of danger signs is a major contributor to delays in seeking obstetric care and hence to high maternal mortality and morbidity.

Informing women about obstetric danger signs is among the strategies designed to enhance the utilization of skilled care whenever obstetrics complications are anticipated [7]. Obstetric danger signs are unexpected obstetric signs that can lead to maternal health complications. These danger signs are mainly classified into three categories. Major danger signs during pregnancy include: severe vaginal bleeding, swollen hands/face, and blurred vision. Major danger signs during labor and childbirth include severe vaginal bleeding, prolonged labor (>12 h), convulsions, and retained placenta. Major danger signs during the postpartum period include severe vaginal bleeding, foul-smelling vaginal discharge, and fever [8].Women’s knowledge about these obstetric danger signs during pregnancy, delivery and postpartum is still low in sub-Saharan African countries evidenced by studies conducted in Burkina Faso [9], Ethiopia [10, 11] and rural Tanzania [12].

Efforts have been made by the Tanzanian government to increase knowledge of obstetric danger signs among women through implementation of focused antenatal care (FANC) in 2002 which provides free counseling on these danger signs to all pregnant women attending to antenatal care (ANC) [13]. The FANC strategy insists ANC providers inform pregnant women about danger signs verbally with the help of visual aids such as brochures and posters. Twelve years since implementation of FANC, only 53% of pregnant women in Tanzania were found to have received information on signs of obstetric complications and about 51% utilized skilled obstetric care [4].

In Chamwino district, part of the Dodoma region, only 48% of pregnant women were informed about obstetric danger signs during ANC visits despite the fact that 98% of women attended ANC at least once [14]. Therefore half of pregnant women in the area are reportedly being counseled about obstetric danger signs. However, the impact of this counseling has not been fully assessed, and the proportion of women who have an adequate level of knowledge about these danger signs and their associated factors is not known.

This study was therefore designed to determine the proportion of recently-delivered women in Chamwino district who had knowledge about obstetrics danger signs and their associated factors. The study additionally assessed whether having knowledge of obstetric danger signs is associated with utilization of skilled birth care during delivery.

## Methods

### Study area

This study was conducted in Chamwino district, Dodoma region, central Tanzania. The district has 3 divisions, 32 wards and 77 villages. It covers an area of 8742 km^2^ with an estimated population of 319,044 in 2013 and a growth rate of about 2.4%. The area is semi-arid, receiving annual rainfall of between 500 and 800 mm. The dominant ethnic group is Gogo who are involved in both crop and livestock production. Per capita income is 180,000/= Tanzania shillings (TSH) per annum. The district has 63 functioning health facilities, including one district-designated hospital, five rural public health centers, 55 government dispensaries, and 2 faith-based dispensaries. It is estimated that in 2013, the district had 77,429 women of childbearing age and a crude birth rate (CBR) of 38.5 births per 1000 population [14, 15].

### Study design and population

A community-based cross-sectional study was conducted among women who delivered within two years prior to data collection regardless of the newborn outcome.

### Sample size and sampling procedure

The sample size was obtained by using the formula for single population proportion. A sample size of 432 women was obtained by employing the following assumptions during sample size calculation: proportion of women who have knowledge on obstetric danger signs was 14.8% [16], level of significance was 95%, margin error was 5%, and non-response rate was 10%.As multistage cluster sampling was used, the calculated sample was multiplied by two for design effects to control for the potential effect of sampling due to using sampling method other than simple random sampling.

A multi-stage cluster sampling procedure was used. At each stage a sampling frame was developed and simple random sampling was employed. First, based on the above calculation, four of the 32 wards in Chamwino District were randomly selected to form a representative sample. From each selected ward, two villages were randomly chosen. Next, one hamlet was randomly chosen from each selected village. Then, the total sample size was allocated proportionally to the size of the selected hamlets. All households with recently-delivered woman in each hamlet were identified with the help of village health workers to create a sampling list. Finally, systematic sampling was used to select the household with recently-delivered woman in each hamlet until the desired number of samples was attained. In each selected hamlet the total number of households with recently-delivered woman was divided by the sample size required for that hamlet in order to calculate the sampling intervals (k).

To select the first household, one of the houses which was included under the initial sampling interval (between 1 and k) of each hamlet was selected by simple random sampling (lottery method). Then, the next household was selected through systematic sampling which was every k^th^ interval household calculated separately for each hamlet (since the total number of households varied from one hamlet to another). In the case that study participants were not available, three attempts were made to find them before they were declared as non-respondents. On the other hand, if the household did not have women who met the inclusion criteria, the next household was substituted. Where there was more than one eligible respondent in a household, one was randomly selected to participate using the lottery method.

Recently-delivered women who were permanent residents of Chamwino District and who were willing to participate and respond to the questionnaire were included in the study. Women who were not permanent residents, not willing to participate in the study, mentally disabled or severely ill were excluded from the study.

### Operational and term definitions

Knowledge of the key obstetric danger signs during pregnancy, childbirth and postpartum: A woman was considered knowledgeable if she spontaneously mentioned at least five danger signs in the three phases with at least one in each phase. Phase 1: Danger signs during pregnancy (vaginal bleeding, swollen hands/face, and blurred vision). Phase 2: Danger signs during labor/childbirth (severe vaginal bleeding, prolonged labor (>12 h), convulsions, and retained placenta). Phase 3: Danger signs during postpartum (severe vaginal bleeding, foul-smelling vaginal discharge, and high fever). This method of scoring has been previously used to assess women’s knowledge of obstetric danger signs [17, 18].

Recently-delivered women: These were defined as women who delivered within two years prior to data collection regardless of the newborn outcome.

Household: This was defined as a group of individuals who ate from the same pot and slept under the same roof in the previous night.

Employment: This was defined as the condition of having paid work. In this study someone was considered as employed or self-employed if he/she had any paid work whether employed by another or self-employed.

Skilled birth attendants: These were defined as people with midwifery skills (physicians, nurses, midwives, and health officers) who can manage normal deliveries and diagnose, manage or refer cases with obstetric complications.

### Data collection tool and procedure

An interviewer-administered questionnaires was used for data collection since 40% of the population of Chamwino District is illiterate [14]. This questionnaire was adapted from a safe motherhood questionnaire developed by the Maternal Neonatal Program of Jhpiego [8] and modified to fit the Tanzanian context. Information regarding socio-demographic characteristics, reproductive characteristics, and knowledge of obstetric danger signs was collected. Five research assistants, who had diplomas in clinical medicine and were fluent in the Swahili language, collected data under the supervision of the principal investigator.

### Data quality control

Data quality was guaranteed by using a validated questionnaire translated by an expert translator from English to the local language (Swahili). Another translator back translated the questionnaire from Swahili to English to check for its original meaning. A pretest was conducted on 5% of the total sample size in the neighboring district of the Dodoma municipality. Research assistants trained for three days, participated in pretesting then conducted interviews under supervision. All questionnaires were counter-checked for completeness and consistency of responses before leaving the field site. Those that were not filled out properly were returned back to the household and filled out correctly.

### Data processing and analysis

Data was entered, cleaned, edited, and coded by using Epi-info version 3.5.1then transferred to STATA version 11.2 for analysis. During descriptive analysis, continuous variables were summarized using mean and standard deviation while categorical variables were summarized using proportions, then presented in tables and graphs. Bivariate analysis was undertaken to test for associations between the dependent variable, knowledge of obstetric danger signs, and the independent variables using Pearson’s chi-square of Fischer’s exact test where appropriate. Then, all variables which showed an association using bivariate analysis (*p*-value <0.2) were fitted into the multiple logistic regression model by the stepwise (forward selection) method to test for the association of each with the dependent variable at the 95% confidence level. The *P*-values and 95% confidence intervals (CI) for odds ratios (OR) were used to confirm significance of the associations. A *P*-value less than 0.05 was considered significantly different. In all our analyses we employed the “svy” set command in STATA to adjust for clustering effect due to complex sampling.

### Ethical considerations

Ethical clearance was requested and approved by Muhimbili University of Health and Allied Sciences (MUHAS) Research Ethics Committee. Permission was also requested and obtained from the Chamwino District executive director, ward executive officers, and village executive officers. The respondents were adequately informed using the participant’s informed consent statement, read aloud by research assistants detailing all relevant aspects of the study, including its aim, interview procedures, anticipated benefits, and potential hazards. Respondents who accepted to participate in the study provided signed written informed consent or thumbprint for those who were illiterate.

## Results

### Socio-demographic characteristics of respondents

A total of 428 out 432 women were recruited in the study yielding a response rate of 99%.The median age (IQR) of the respondents was 26.5 [22–33] years. Among all respondents 119 (27.8%) had no formal education. Only 74 (17.3%) of the respondents were employed through contractual work or self-employment. The majority of respondents, 333 (77.8%) were currently living with their partner i.e. married or cohabiting (Table 1).

**Table 1:** Socio-demographic characteristics of the respondents, Chamwino district, Tanzania, January, 2014 (*N* = 428)

Variable	Frequency	Percentage
Age years (Median 26.5, Range 16.0,50.0)		
< 20	41	9.6
20–29	227	53.1
30–39	135	31.5
≥ 40	25	5.8
Marital status		
Single	70	16.4
Married/Cohabiting	333	77.8
Widow/Separated/Divorced	25	5.8
Educational status		
None	119	27.8
Primary	286	66.8
Secondary or above	23	5.4
Occupation		
Employed/Self-employed	74	17.3
Not employed	354	82.7
Spouse’s education status^a^		
None	53	15.9
Primary	254	76.3
Secondary or above education	26	7.8
Spouse’s occupation^a^		
Employed/Self-employed	52	15.6
Not employed/Peasant	281	84.4
Monthly income (TSH)^b^		
< 50,000/=	381	89.0
50,000–100,000/=	40	9.4
> 100,000/=	7	1.6

^a^Could not add up to 428 because some of the respondents did not have spouse at the time of survey

^b^1 US dollar = 2, 100 TSH

### ANC and obstetric history of the respondents

All of the respondents reported having attended ANC at least once during their last pregnancy. The majority of respondents (*n* = 316, 73.8%had attended at least four ANC visits (the minimum recommended number of visits) while 50 (11.7%) had attended only one. Only a few of the respondents (*n* = 74, 17.3%) had booked an ANC visit during the first trimester of their pregnancy. A total of 363 (84%) respondents reported that they received counseling on obstetric danger signs.

A total of 292 (68.2%) of women received skilled obstetric care during delivery. Of those who received skilled care, the majority (52.7%; 154/292) delivered in dispensaries while 20.8% (61/292) delivered in a hospital (Table 2).

**Table 2:** Antenatal and obstetrics history among recently-delivered women, Chamwino district, Tanzania, January, 2014 (*N* = 428)

Variable	Frequency	Percentage
Gestational Age at booking		
1^st^ trimester	74	17.3
2^nd^ trimester	336	78.5
3^rd^ trimester	18	4.2
No. of ANC visits (Mean = 3.7, SD = 0.6)		
1 visit	50	11.7
2–3 visits	62	14.5
≥ 4 visits	316	73.8
Counseling about danger signs		
Counseled	363	84.8
Not counseled	65	15.2
Parity (Mean = 3.2, SD = 1.9)		
1	108	25.2
2–4	208	48.6
≥ 5	112	26.2
Skilled obstetric care		
Yes	292	68.2
No	136	31.8

### Respondents’ knowledge of obstetric danger signs

A total of 108 (25.2%) respondents, spontaneously mentioned severe vaginal bleeding, 88 (20.6%) mentioned blurred vision, and 86 (20.1%) mentioned swollen hands/face as the key danger signs during pregnancy. A total of 119 (27.8%), 73 (17.1%), 60 (14.0%) and 68 (15.9%) respondents, spontaneously mentioned severe vaginal bleeding, retained placenta, prolonged labor, and convulsions as key danger signs during childbirth or labor respectively. Of all respondents, 111 (25.9%), 65 (15.2%), and 61 (14.3%) spontaneously mentioned severe vaginal bleeding, high fever, and foul-smelling vaginal discharge as danger signs during the postpartum period, respectively (Table 3).

**Table 3:** Knowledge of obstetrics danger signs among recently-delivered women, Chamwino district, Tanzania, January, 2014 (*N* = 428)

Variable	Frequency	Percentage
Key danger signs during pregnancy		
Severe vaginal bleeding	108	25.2
Swollen hands/face	86	20.1
Blurred vision	88	20.6
Key danger signs during delivery		
Severe vaginal bleeding	119	27.8
Retained placenta	73	17.1
Labour lasting more than 12 h	60	14.0
Convulsions/fits	68	15.9
Key danger signs during postpartum		
Severe vaginal bleeding	111	25.9
High fever	65	15.2
Foul smelling vaginal discharge	61	14.3
Number of obstetrics danger signs reported		
Did not mention any danger signs	294	68.7
Mentioned 1–4 danger signs	26	6.1
Mentioned at least 5 danger signs	108	25.2

Note: The frequency and percentage cannot add up to 428 and 100% respectively because multiple responses were possible

### Score on knowledge of obstetric danger signs

**Fig. 1:**
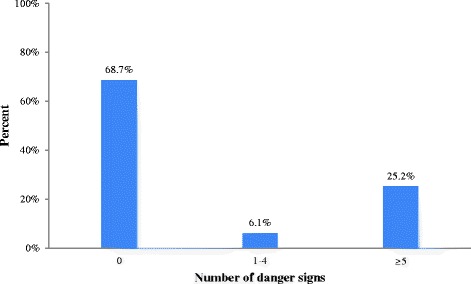
Number of obstetrics danger signs reported among recently-delivered women, Chamwino district, Tanzania, January, 2014 (*N* = 428)

A total of 294 (68.7%) of the respondents were not able to mention danger signs in any of three phases while 134 (31.3%) mentioned at least one of the key danger signs. Only 108 (25.2%) of respondents were able to mention at least five key danger signs in the three phases and thus were regarded as having knowledge of key danger signs during pregnancy, childbirth/labor and postpartum (Fig. 1).

### Factors associated with knowledge of obstetric danger signs

In bivariate analysis maternal age, maternal education, maternal occupation, spouse occupation, and counseling on danger signs were found to have statistically significantly associations with knowledge of obstetric danger signs.

In multiple logistic regression, the initial model included knowledge of obstetric danger signs as the outcome variable while maternal age, maternal education, maternal occupation, spouse occupation, counseling on danger signs, and monthly income were included as explanatory variables. The final model revealed that the odds of knowledge of obstetric danger signs were two times higher among women who had primary education and above compared to those with no education (AOR = 1.96; 95% CI: 1.01, 3.82). Also the odds of knowledge of obstetric danger signs were two times higher among employed women compared to unemployed women (AOR = 2.23; 95% CI: 1.10, 4.52). Additionally, women with an employed spouse were found to have two times higher odds of knowledge of obstetric danger signs compared to their counterparts without an employed spouse (AOR = 2.10; 95% CI: 1.02, 4.32). Furthermore, the odds of knowledge of obstetric danger signs were three times higher among women who were counseled on danger signs during ANC visits compared to those who were not (AOR = 3.42; 95% CI: 1.36, 8.62) (Table 4).

**Table 4:** Socio-demographic, obstetric and antenatal care factors predicting knowledge of obstetric danger signs among recently-delivered women, Chamwino district, Tanzania, January, 2014 (*N* = 428)

Variable knowledge on obstetrics danger signs			COR (95%: CI)	AOR (95%: CI)
	Yes *N* (%)	No *N* (%)		
Age				
< 20	5 (04.63)	36 (11.25)	0.38 (0.15, 0.97)	0.30 (0.07, 1.32)
≥ 20	103 (95.37)	284 (88.75)	1.00	
Marital status				
Living with spouse	88 (81.48)	244 (76.25)	1.37 (0.79,2.36)	
Not living with spouse	20 (18.52)	76 (23.75)	1.00	
Educational status				
Primary and above	91 (84.26)	108 (08.13)	2.50 (1.43,4.40)	1.96 (1.01,3.82)
No education	17 (15.74)	102 (31.87)	1.00	1.00
Occupation				
Employed	32 (29.63)	42 (13.12)	2.79 (1.65,4.70)	2.23 (1.1, 4.52)
Others	76 (70.37)	278 (86.88)	1.00	1.00
Spouse’s educational status				
Employed	9 (16.98)	79 (28.21)	0.52 (0.25, 1.10)	
Unemployed	44 (83.02)	201 (71.79)	1.00	
Spouse’s occupation status				
Employed	25 (28.41)	27 (11.02)	3.20 (1.75,5.88)	2.10 (1.02, 4.32)
Unemployed	63 (71.59)	218 (88.98)	1.00	1.00
Monthly income (Tsh)				
≥ 50,000/=	16 (14.81)	31 (09.69)	1.62 (0.86,3.08)	0.98 (0.45, 2.11)
< 50,000/=	92 (85.19)	289 (90.31)	1.00	1.00
Parity				
1	23 (21.30)	85 (26.56)	0.75 (0.45, 1.26)	
≥ 2	85 (78.70)	235 (73.44)	1.00	
No. of ANC visits				
1 visit	13 (12.04)	37 (11.56)	1.00	
2–3 visits	13 (12.04)	49 (15.31)	0.75 (0.31, 1.82)	
≥ 4 visits	82 (75.92)	234 (73.13)	1.01 (0.51, 2.00)	
Counseling about danger signs				
Yes	100 (92.59)	263 (81.88)	2.77 (1.30, 5.90)	3.42 (1.36, 8.62)
No	8 (07.41)	57 (18.12)	1.00	1.00

## Discussion

This community survey assessed the knowledge of obstetric danger signs and associated factors among recently-delivered women in the Chamwino District, Tanzania. Having knowledge of obstetric danger signs is an essential step in recognizing complications and enables one to take appropriate action to access emergency care [19]. The current study found that knowledge of obstetric danger signs among women in Chamwino district was not very prevalent despite the fact that the majority of them stated that they were counseled about obstetric danger signs during ANC visits. The possible reason for this might be that counseling offered during ANC visits was not of good quality or because women received this counseling in a group and not at the individual level. Also, previous studies observed a short (less than three minutes) time spent for individual counseling [12, 20, 21] compared to 15 min as recommended by FANC simulation conducted in Tanzania [21]. This low prevalence of knowledge about danger signs was reported also in previous studies done in Mpwapwa, Tanzania, rural Uganda, Egypt and Ethiopia [16, 22–25]. This might be due to similar socio-economic characteristics in these study settings.

Among obstetric complications, almost a quarter of global maternal deaths are due to vaginal bleeding during pregnancy, childbirth and postpartum [26, 27]. In this study danger signs were classified into three phases, and in each phase severe vaginal bleeding was the most commonly mentioned danger sign in line with studies undertaken in sub-Saharan countries [11, 12, 23, 28]. This may be due to women thinking that, as opposed to some of more equivocal signs, vaginal bleeding is perceived as clearly abnormal.

It is estimated that about 10% of pregnancies worldwide are accompanied by preeclampsia, the second leading cause of direct maternal death. In this study we found that a high proportion of women failed to mention danger signs associated severe preeclampsia and eclampsia (i.e. swelling of hands/face, blurred vision and convulsions). These findings are consistent with other studies conducted in Robe Wareda, Ethiopia [29] and Mulago Hospital, Uganda [30]. This may due to inadequate emphasis on informing women about all obstetric danger signs during ANC, as our study shows that the majority of the respondents (74%) had attended at least four ANC visits during their last pregnancy. Similarly, the current study shows that there is no difference about knowledge of obstetrics danger signs as related to number of ANC visits. This is in agreement with findings from study done in Ethiopia which found no association between number of ANC visits and knowledge of danger signs [31]. However, other previous studies have shown a significant association [12, 32, 33]. Despite these contradictory findings, we still believe that more number of ANC visits are important for pregnant women not only to be advised about danger signs but also to receive maternal health education as whole.

Women who completed at least primary education were more likely to be knowledgeable about obstetric danger signs compared to those with no formal education. This finding is consistent with previous studies done in Tsegedie District, Tigray Region, Ethiopia [33], KwaZulu-Natal [34], and Jordan [35]. This might be because educated women may tend to be have more autonomy in making decisions about issues related to their own health, and to be more empowered in accessing the health service information needed to act on ANC advice about obstetric danger signals.

Employed women were more likely to be knowledgeable about obstetric danger signs compared to unemployed women. This is in line with a similar study conducted in Goba District, Ethiopia [28]. This could be because women who earn their own salaries might be more autonomous when seeking better health care compared to unemployed women.

Women whose spouses were employed were more likely to be knowledgeable about obstetric danger signs compared to women whose spouses were not employed at the time of the survey. This might be because living with an employed spouse means a greater likelihood of having cash to access and utilize health services like ANC, which aims to educate women about obstetric danger signs.

Counseling about obstetric danger signs is among the fundamental functions of ANC which aims to reduce the delay in seeking obstetric care in case of any obstetric complications. All pregnant women in Tanzania are expected to discuss danger signs with ANC providers. In this study we found that women who received counseling about obstetric danger signs during ANC visits were more likely to be knowledgeable about obstetric danger signs compared to those who did not receive counseling. Similar findings were reported from previous study done in Tanzania [12]. The similarity of this finding observed might be explained by the similarity of study participants (delivered women) and study settings (rural district). Also, women who were informed about danger signs during ANC tended to have better awareness of warning signs of obstetric complications.

Knowledge of obstetric danger signs is essential for encouraging women and their families to seek skilled health care in case of complications either before or during delivery. In this study we found that women who were knowledgeable about obstetric danger signs were more likely to utilize skilled birth attendants during delivery compared to those who were not knowledgeable. However, the current study did not collect information about previous obstetric complications. Therefore, we could not control for this during our analysis; this factor might confound the observed association between knowledge of obstetrics danger signs and utilization of skilled birth attendants during delivery.

The strength of the study is that we used a community-based survey in order to minimize the effect of selection bias. Cluster sampling was employed in order to obtain a representative sample. Finally we used trained health personnel during data collection. Subjects in the same cluster may have shared similar characteristics; this could have distorted our results by either neutralizing or overestimating the prevalence or association. This distortion was minimized by adjusting for clustering during data analysis. The major limitations of this study were due to nature of cross-sectional surveys, the temporal relationship could not be established and recalls bias such that those women who suffered complications may be more likely to recall danger signs than those who did not suffer from complications. We attempted to minimize recall bias by including women who delivered within two years prior to survey.. Also, this study did not assess the source of information about obstetric danger signs. Hence it is hard to say whether women received the knowledge from ANC or elsewhere such as through magazine, radio, television or personal experience.

## Conclusion

The findings from this study showed a low prevalence of knowledge about obstetric danger signs among women in the study area. Significant factors associated with knowledge about obstetric danger signs were maternal education and occupation, spouse occupation and counseling about obstetric danger signs during ANC. District education officers should put efforts towards empowering women with education since maternal education was found to be associated with having knowledge about obstetric danger signs. It seems that educated women can retain the information received during ANC visit. Counseling about obstetric danger signs was found to be significantly associated with having knowledge of obstetric danger signs despite the fact that majority were counseled yet, only few were knowledgeable about obstetric danger signs. Therefore, we recommend the ministry of health to design and distribute the maternal health booklets that highlight the obstetric danger signs and encourage antenatal care providers and community health workers to provide frequent health education about these danger signs for all pregnant women. Also, ANC providers should improve the quality of counseling about obstetric danger signs and ensure that, every pregnant woman during ANC visit receive this counseling. In this study, we did not assess the quality of counseling about obstetrics danger signs. Therefore further research should be conducted to assess whether the quality of counseling provided to pregnant women explains the level of knowledge about obstetric danger signs.

## Abbreviations

ANC: Antenatal care
AOR: Adjusted odds ratio
COR: Crude odds ratio
FANC: Focused antenatal care
MMR: Maternal mortality ratio
TSH: Tanzania shillings
